# Comparison of Outcomes Between Open Major Hepatectomy Using CUSA and Laparoscopic Major Hepatectomy Using “Lotus” Liver Blade. A Propensity Score Matched Analysis

**DOI:** 10.3389/fsurg.2019.00033

**Published:** 2019-05-31

**Authors:** Minas Baltatzis, Ahmed Mirza, Panos Stathakis, Ahmed Tyurkylmaz, Saurabh Jamdar, Ajith K. Siriwardena, Aali J. Sheen

**Affiliations:** ^1^Regional Hepato-Pancreato-Biliary Unit, Manchester Royal Infirmary, Manchester, United Kingdom; ^2^Faculty of Biology, Medicine and Health, University of Manchester, Manchester, United Kingdom; ^3^Department of Biomedical Research Manchester Metropolitan University, Manchester, United Kingdom

**Keywords:** liver cancer, major hepatectomy, laparoscopy, propensity score matching analysis, Lotus energy device

## Abstract

**Introduction:** Evolution in laparoscopic liver surgery during the past two decades is an indisputable fact. According to the second international consensus conference for laparoscopic liver resection held in Morioka, Japan in 2014 major resections are still regarded as innovative procedures in the exploration phase. On this basis, our study aims to explore the efficacy and safety of laparoscopic vs. open major liver resection and therefore increase the existing evidence on major laparoscopic liver surgery.

**Methods:** All consecutive patients who underwent major liver resection, open and laparoscopic from January 2016 to May 2018 were identified from our prospectively maintained database. Propensity score matching analysis was performed using R statistical tool in SPSS to isolate matched open and laparoscopic cases which were compared for intraoperative and postoperative short-term outcomes. Lotus ultrasonic energy device was used for parenchymal transection in laparoscopic cases vs. CUSA in open procedures.

**Results:** Propensity score matching analysis was performed on 82 consecutive patients (61 open and 21 laparoscopic major hepatectomies) resulting in 40 matched patients, 20 in each group. The mean total duration of surgery and duration of parenchymal transection were slightly longer in the laparoscopic group (*p* = 0.419, *p* = 0.348). There was no difference in the intraoperative and postoperative transfusion rates. Patients after laparoscopic surgery were discharged 2 days earlier on average (*p* = 0.310). No difference was observed in complication rates and mortality.

**Conclusion:** Our data did not reveal inferiority of the laparoscopic major hepatectomy vs. the open approach in any parameter compared. The use of the Lotus ultrasonic energy device appeared to be efficient and safe for parenchymal transection in the laparoscopic procedures.

## Introduction

Laparoscopic partial hepatectomy for liver tumor was first reported by Gagner in 1992 (1). Since that first description, the technique has become progressively more frequently incorporated into routine clinical practice (2). The benefits of laparoscopy for abdominal surgery appear to translate to laparoscopic liver surgery with evidence of less post-operative pain, earlier mobilization, shorter hospital stay and better quality of life being seen after laparoscopic hepatectomy when compared to the open operation (3). Two international consensus conferences for laparoscopic liver resection (LLR) in 2009 (Louisville, USA) in 2014 (Morioka, Japan) have promoted guidelines for the safe adoption of the minimally invasive approach to liver resection (4, 5). The Morioka consensus conference did however state that the laparoscopic approach remains in the exploratory phase for major hepatectomy (5). These concerns relate to the potential difficulties encountered in undertaking the procedural steps of major open hepatectomy including mobilization of the liver from the inferior vena cava, inflow and outflow control by laparoscopy (6, 7). A more recent consensus conference, held in Southampton, UK in 2017, stated that in experienced hands, laparoscopic hemi-hepatectomies are associated with reduced hospital stay and blood loss. The experts also suggested that the feasibility, reproducibility, and implementation of left and right hepatectomies are sufficiently different that they should be considered separately (8).

Recent advances in laparoscopic surgery such as the introduction of new liver transection equipment and the availability of newer haemostatic agents have considerably improved the facility of undertaking major laparoscopic hepatectomy (9–11). The majority of published reports describe laparoscopic left lateral sectionectomy and excision of peripherally located liver lesions in segments IVb, V and VI (3, 12, 13). Data on major laparoscopic liver resections are based on case series and on recently published experience based guidelines (14, 15), but level 1 data are awaited.

The vast majority of reported cases have shown that laparoscopic parenchymal transection is feasible using the modern techniques utilized in open surgery such as the Cavitron ultrasonic surgical aspirator (CUSA), bipolar compression devices and ultrasonic energy devices (16–18). According to a comprehensive review of the literature conducted in the 2nd International Consensus Conference on Laparoscopic Liver Resection, hepatobiliary surgeons should select techniques based on a sound understanding of instruments to be used (19).

The aim of this study was to report the implementation of laparoscopic major hepatectomy in our tertiary center and compare the intraoperative and postoperative outcome between open and laparoscopic major hepatectomy using the Lotus Ultrasonic energy device (BOWA-electronic GmbH, Gomaringen, Germany) for liver parenchymal transection in laparoscopic surgery. Propensity score matching was used to deliver to groups who were comparable in terms of demographic profile, disease distribution and extent of surgery and therefore to reduce patients' selection bias.

## Patients and Methods

### Study Design

This is a single center clinical cohort study based on retrospective analysis of prospectively collected data on patients who underwent major liver resection by either the open or laparoscopic routes in the tertiary regional hepato-pancreato-biliary center of the Manchester Royal Infirmary during the period January 2016 to May 2018. It is based on a consecutive series of patients operated by three consultant hepatobiliary surgeons. Patients were identified from databases maintained prospectively by the three surgeons. Only those with histological confirmation of malignant liver disease, primary or metastatic, were included. Liver resections performed for benign conditions were excluded. Open resections for perihilar cholangiocarcinomas were also excluded, as there were no comparators in the laparoscopic group. Propensity score matching analysis was performed in the original sample, which resulted in a smaller number of matched open and laparoscopic cases, which were used for comparison.

### Definitions

Major open hepatectomy is defined as resection of four or more liver segments, using the Brisbane terminology (20). The definition of laparoscopic major hepatectomy was originally proposed in the Louisville international consensus statement for laparoscopic liver surgery (USA 2008) (4) and confirmed in the second international consensus conference held in Morioka, Japan in 2015 (5). According to these statements the term laparoscopic major hepatectomy includes hemihepatectomies, trisectionectomies and resections of the posterior superior segments (IVa, VII, VIII).

### Pre-operative Assessment

All patients underwent standard pre-operative evaluation. Staging computed tomographic (CT) scan of thorax, abdomen and pelvis and contrast-enhanced magnetic resonance (MR) scans of the liver were performed routinely.^18^Fluoro-deoxyglucose positron emission tomography FGD-PET) scans and preoperative tumor markers were utilized selectively. Eligibility for resection based on the pre-operative work-up was discussed at the regional Hepatobiliary multidisciplinary team meeting (MDT). Selection between laparoscopic vs. open approach was made according to the MDT recommendation and surgeons' personal preference and skills. All eligible patients aged above 60 with or without co-morbidities or above 50 with an underlying co-morbidity underwent cardio-pulmonary exercise test to assess fitness for surgery.

### Surgical Technique

#### Open Hepatectomy

General anesthesia with arterial and central venous pressure monitoring was used. Epidural anesthesia was used for post-operative pain relief. Access was via an epigastric midline incision with right transverse extension. Low central venous pressure anesthesia was used during parenchymal transection. Intra-operative ultrasound was used in all the cases to confirm the preoperative findings, mark the area of transection and to ensure that all the lesions were removed. The cavitron ultrasonic suction aspirator (CUSA, Valleylab, Offaly, Ireland) was used for hepatectomy together with vascular staplers for control of major pedicles intrahepatically. No extra-glissonian vascular dissection was performed.

#### Laparoscopic Hepatectomy

General anesthesia with arterial and central venous pressure monitoring was used. In terms of positioning the left lateral position was used for mobilization of the right lobe of the liver in laparoscopic right hepatectomy followed by repositioning to lithotomy position for parenchymal transection. Laparoscopic left hepatectomy was performed with the patient in lithotomy position throughout the whole procedure. A maximum of two 12 mm trocars and two to three 5 mm trocars were used for the laparoscopic cases with injection of local anesthesia at port sites being utilized for post-operative pain control. Intraoperative USS was used as per open surgery. The Lotus Ultrasonic energy device with a specially designed liver blade was used for parenchymal transection. Vascular staplers were used for major pedicle control intrahepatically, which is similar to the technique used in open surgery.

### Data Collection

Data from the three surgeons' databases were merged in one electronic spread sheet by three co-authors (MB, AM, and AT) and were split into 4 main categories: (a) demographic details (age, gender, World Health Organization performance status score), (b) disease related parameters (histological diagnosis, unilobar/bilobar liver disease, neoadjuvant chemotherapy details), (c) surgical procedure details (type of resection, duration of surgery, duration of Pringle maneuver, parenchymal transection time, transfusion rate), and (d) outcome (resection margin status, postoperative morbidity using Clavien-Dindo classification, hospital stay, 30-day readmission and mortality).

### Ethics

The study was categorized as an audit by the Manchester Hospitals Foundation Trust Research and Development office and was registered with the hospital's audit department. Ethics committee approval was sought and regarded as not required as per a decision made by a trust research committee after using the NHS Health Research Authority (hra) decision toolkit.

### Statistical Analysis

Propensity score matching analysis was performed using the R statistical tool for SPSS (IBM Corp; IBM SPSS Statistics for Windows, Version 23.0, Armonk, NY, USA). Nearest neighbor was the matching algorithm used in our analysis, with a match ratio of 1:1. Caliper value was set to 0.2. Patients' characteristics selected for the matching analysis were the following: age, administration of chemotherapy prior to resection, colorectal liver metastases vs. other malignancies, disease distribution (unilobar vs. bilobar) and WHO performance status score. Outcome comparisons were performed using One-Way ANOVA and Chi-Square test in SPSS. Statistical significance was defined as *p* < 0.05.

## Results

### Study Characteristics

Between January 2016 and May 2018, 82 patients underwent major hepatectomy under the care of the three HPB consultants. 61 of them (74%) had open major hepatectomy and 21 laparoscopic (26%). Histological diagnoses were colorectal liver metastases (CRLM) in 54 patients (66%), hepatocellular carcinoma in 7 (8%), renal metastases in 4 (5%), breast metastases in 3 (4%), metastatic melanoma in 2 (2%), intrahepatic cholangiocarcinoma in 2 (2%), sarcoma in 2 (2%), metastatic meningioma, cystadenocarcinoma, gallbladder cancer and metastatic gastrointestinal stromal tumor (GIST) in 1 patient each. Surgical procedures performed were hemihepatectomies (69 patients, 84%), trisectionectomies (6 patients, 7%) and laparoscopic major resections including posterior segments (4a, 7,8), as per Louisville consensus definition (7 patients, 9%).

### Propensity Score Matching Analysis

**Figure 1:**
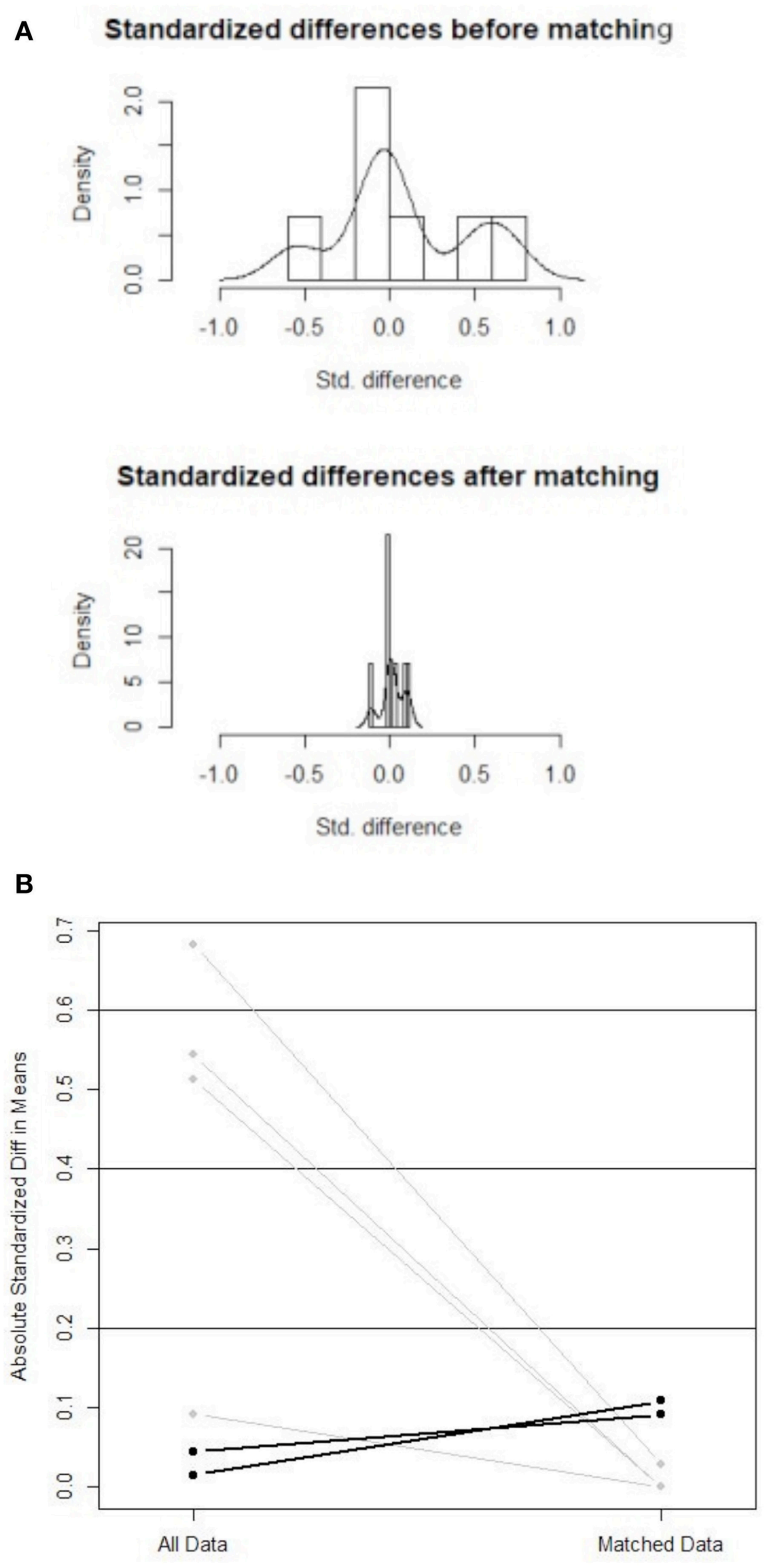
**(A)** Standardized differences within the sample before and after the matching process. **(B)** Absolute standardized difference in means (comparison of all data with matched data).

Propensity score matching analysis resulted in 20 open and 20 laparoscopic matched cases, which comprise the study population used for comparisons. Table 1 summarizes the results of the matching analysis. Figure 1A demonstrates the decreased standardized differences within the sample after the matching process. Figure 1B shows lower absolute standardized difference in the matched data compared with the original data. Median age, WHO performance status score, disease diagnosis and distribution as well as administration of chemotherapy prior to resection were compared within the matched open and laparoscopic groups to evaluate the accuracy of the matching process. No statistically significant difference was observed in any of the above parameters, as demonstrated in Table 2.

**Table 1:** Propensity score matching analysis—summary.

**Subsamples**	**All**		**Matched**		**Unmatched**		**Discarded**	
	**Open**	**Laparoscopic**	**Open**	**Laparoscopic**	**Open**	**Laparoscopic**	**Open**	**Laparoscopic**
N	61	21	20	20	41	1	0	0
**Total**	**82**		**40**		**42**		**0**	

**Table 2:** Comparison of clinical characteristic between the open and laparoscopic groups after the matching process.

	**Open**	**Laparoscopic**	***P*-value**
Age (median-range)	60 (33–88)	64 (32–80)	0.644
WHO^†^ performance status (median-range)	0 (0–1)	0 (0–1)	1.000
Unilobar/bilobar disease (*n*)	15/5	15/5	1.000
Neoadjuvant chemotherapy (*n*)	11	11	1.000
CRLM^*^/other malignancies (*n*)	10/10	14/6	0.206

† WHO, World Health Organization.

* *CRLM, colorectal liver metastases*.

### Short Term Outcome Comparison

The mean duration of the laparoscopic liver resections (mean±SD = 271 ± 107.8 minutes) was slightly longer than in the open resections (247 ± 74.5 min), *p* = 0.419. Pringle maneuver was 59 min in laparoscopic procedures compared to 41 min in open operations (*p* = 0.062). The duration of parenchymal transection was longer in laparoscopic hepatectomies (*p* = 0.348). Four patients (20%) required intraoperative transfusion in the open group and 2 (10%) in the laparoscopic group (*p* = 0.698).

**Figure 2:**
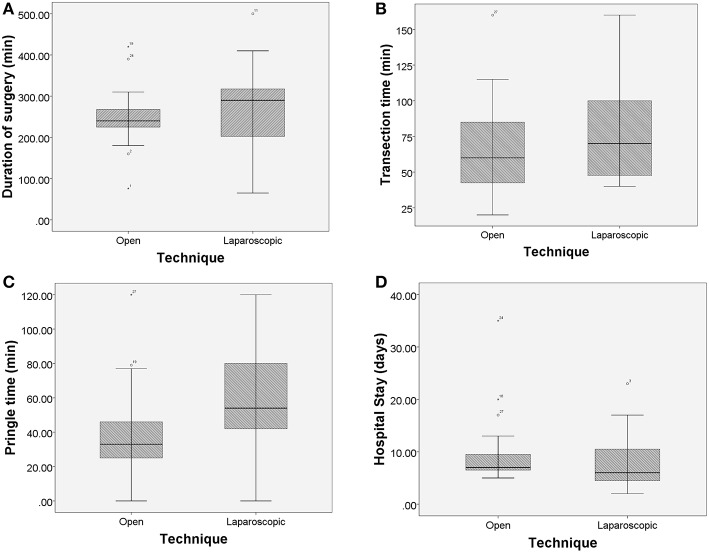
**(A–D)** Boxplot graphs demonstrating comparison of surgical times and inpatient stay with 95% confidence intervals. An asterisk represents an extreme outlier (a value more than 3 times the interquartile range from a quartile). A circle is used to mark other outliers with values between 1.5 and 3 box lengths from the upper or lower edge of the box.

During the postoperative period 2 patients were transfused after open hepatectomy and none after laparoscopic, without this finding being statistically significant. There was also no difference between the groups in the rate of mild complications (Clavien-Dindo I and II) and serious complications (Clavien-Dindo III and IV). The total complication rate was the same (30%) in both groups. 6 patients in total were complicated with bile leak, 3 in each group. There were no post-operative deaths. In terms of postoperative inpatient stay, patients after laparoscopic surgery were discharged 2 days earlier on average (approximately 8 days in the laparoscopic group vs. 10 days in the open group, *p* = 0.310). Histological examination of the specimens confirmed negative resection margin in 55% of the open procedures and 80% of the laparoscopic procedures, without this difference being statistically significant (*p* = 0.096). There was also no difference in the 30-day readmission rates between the groups. Table 3 tabulates the surgical procedures carried out in the matched study population. Table 4 summarizes the comparative analysis between the open and laparoscopic groups. Figures 2A-D demonstrate boxplot graphs of the numerical variables analyzed, including 95% confidence intervals.

**Table 3:** Surgical procedures performed in the matched study population.

	**Open**	**Laparoscopic**	**Total**
Right hepatectomy^*^	14	14	28
Left hepatectomy^†^	5	2	7
Right posterior sectionectomy^‡^	0	4	4
Left trisectionectomy	1	0	1

* 3 open and 4 laparoscopic right hepatectomies were combined with contralateral metastasectomies.

† *1 open left hepatectomy was combined with segment 5 metastasectomy*.

‡ *1 Right posterior sectionectomy was combined with left lateral sectionectomy*.

**Table 4:** Comparison of the intraoperative and postoperative outcome between the open and the laparoscopic groups.

	**Open**	**Laparoscopic**	***P*-value**
**Duration of surgery** (min, mean ± SD)	247.6 ± 74.5	271.5 ± 107.8	0.419
**Duration of pringle maneuveur** (min, mean ± SD)	40.9 ± 28.2	59.3 ± 29.8	0.062
**Parenchymal transection time** (min, mean ± SD)	69.3 ± 35.5	82.0 ± 42.2	0.348
**Transfusion**			
Intraoperative (*n*, %)	4/20 (20%)	2/20 (10%)	0.698
Postoperative (*n*, %)	2 (10%)	0	0.178
**Complications**			
Clavien-Dindo I+II (*n*, %)	3/20 (15%)	3/20 (15%)	1.000
Clavien-Dindo III+IV (*n*, %)	3/20 (15%)	3/20 (15%)	1.000
Bile leak	3/20 (15%)	3/20 (15%)	1.000
Total (*n*, %)	6/20 (30%)	6/20 (30%)	1.000
**Mortality (*****n*****, %)**	0	0	1.000
**R0 resection margin (*****n*****, %)**	11/20 (55%)	16/20 (80%)	0.096
**Hospital stay** (days, mean ± SD)	9.9 ± 7.1	7.9 ± 5.4	0.310
**30-day readmission (*****n*****, %)**	1/20 (5%)	2/20 (10%)	0.095

## Discussion

After the first report of a laparoscopic wedge liver resection in 1991 (13), laparoscopic liver resection has been explored increasingly, especially the last decade. However, only a small percentage of liver resections are performed laparoscopically, according to a recent multicentre study (6.7%) (21) and a French national survey (17.8%) (22). The vast majority of patients still undergo smaller resections rather than major hepatectomies with some feasibility studies demonstrating the increased operating time required but a definite trend of a reduced hospital stay (23). Undertaking the steps to accommodate laparoscopic liver surgery in any unit must rely on a team approach as well as high volume of cases with good experience in surgical ability (15, 24).

Whilst the surgical fraternity awaits the outcome of the ORANGE-II trial, which will provide the necessary level 1 data (25), there are a plethora of data suggesting that laparoscopic liver surgery is here to stay (26, 27). A large propensity matched analysis from Japan including 531 matched individuals showed the laparoscopic arm depicting a reduced median post-operative stay (12 days vs. 14 days; P < 0.001). In this study the exact transection techniques were not described but it appears that some of the operations were carried out using non-pure laparoscopic approaches including a hybrid approach. Another propensity score matching analysis (153/153 patients matched) demonstrated a reduction in hospital stay (11.1 vs. 13.9 days) as well as reduced rates of serious complications (21). Comparable results are also demonstrated in large case series comparison between open and laparoscopic surgery (28). Results from a meta-analysis of seven observational studies including 624 patients revealed a lower incidence of R1 resections in the laparoscopic resections which is contrary to previous skeptical views on the laparoscopic approach as well as less blood loss and transfusion requirements (29).

Although a Propensity score matching analysis is recognized as comparable to a randomized trial (30), this study still has limitations. The limitations include the relatively small number of patients resulting in a low statistical power as well as the heterogeneity of patients' diagnosis. The low numbers may have contributed to the negative result of the matched propensity scoring despite a leaning toward the LOTUS energy device. As a result of the latter the study focuses on the short-term outcome analysis only.

Accepting the above limitations, this is the first study on major laparoscopic liver surgery vs. open surgery using a propensity score matching analysis, with liver transection in the laparoscopic arm undertaken by a specially designed laparoscopic ultrasonic liver blade (LOTUS). Our data suggest that the introduction of major laparoscopic liver surgery undertaken predominantly by the most experienced surgeons in our unit has had favorable outcomes, a fact shadowed by publications from other centers, which highlight that surgeons experience is a foremost requirement for the use of minimal access techniques in liver surgery (24, 31, 32). This preliminary report also shows that the use of the specially designed liver blade for the laparoscopic cases did not add any feasibility or safety concerns. This fact distinguishes to a degree and separates this study from other comparable reports using the traditional translation of the open techniques to laparoscopic surgery.

Furthermore, our data reveal that there was no difference in morbidity and blood transfusion rates. Although not statistically significant, there was a leaning toward a reduced amount of blood loss in the laparoscopic arm. Patients on the laparoscopic arm had a shorter hospital stay by 2 days compared to the open surgery arm, which reflects the globally observed decrease of inpatient stay after any type of laparoscopic surgery compared to the equivalent open procedures (33). The prolonged operating and pringle time in the laparoscopic arm may be attributed to the steep learning curves rather than to the technique itself (31, 32).

The results of this study, although not statistically significant, are comparable with a recently published meta-analysis including 5,889 patients from 47 studies. According to this meta-analysis laparoscopic hepatectomies were associated with less operative blood loss, lower blood transfusion requirement, higher R0 resection rate and shorter hospital stay (34). Our similar results for major hepatectomies exclusively are suggestive that laparoscopic major liver resection might become the standard practice in the near future provided that the awaiting results of ORANGE-II randomized trial will confirm the above findings.

In conclusion, our data show that laparoscopic major hepatectomy does not seem to be inferior to the open approach in any aspect. The use of the Lotus ultrasonic energy device with the specially designed liver blade appears to be efficient and safe, as no increase in postoperative morbidity, especially bile leaks and mortality was observed. In the future it maybe of value to further report data utilizing this technique with a larger sample of patients?

## Ethics Statement

The study was categorized as an audit by the Manchester Hospitals Foundation Trust Research and Development office and was registered with the hospital's audit department. Ethics committee approval was sought and regarded as not required as per a decision made by a trust research committee after using the NHS Health Research Authority (HRA) decision toolkit.

## Author Contributions

AJS provided patients' database, conceived the idea, supervised the manuscript writing. AKS provided patients' database, supervised the manuscript writing. SJ provided patients' database. MB, AM, AT, and PS data extraction. MB Statistical analysis, methods, and results.

### Conflict of Interest Statement

The authors declare that the research was conducted in the absence of any commercial or financial relationships that could be construed as a potential conflict of interest. The reviewer CM declared a shared affiliation, with no collaboration within the last two years, with the authors to the handling editor at the time of review.
